# Mechanism of Drug Resistance to First‐Line Chemotherapeutics Mediated by TXNDC17 in Neuroblastomas

**DOI:** 10.1002/cnr2.70033

**Published:** 2024-10-16

**Authors:** Chenggong Zeng, Zhuoran Li, Zhiqing Wei, Tingting Chen, Juan Wang, Junting Huang, Feifei Sun, Jia Zhu, Suying Lu, Zijun Zhen

**Affiliations:** ^1^ State Key Laboratory of Oncology in South China, Guangdong Provincial Clinical Research Center for Cancer Sun Yat‐Sen University Cancer Center Guangzhou PR China; ^2^ Collaborative Innovation Center of Cancer Medicine Sun Yat‐Sen University Cancer Center Guangzhou PR China; ^3^ Department of Pediatric Oncology Sun Yat‐Sen University Cancer Center Guangzhou PR China

**Keywords:** autophagy, BECN1, drug resistance, Neuroblastoma, TXNDC17

## Abstract

**Background:**

The prognosis of high‐risk neuroblastomas (NB) that are resistant to first‐line induction chemotherapy is relatively poor. This study explored the mechanism of resistance to first‐line chemotherapeutics mediated by TXNDC17 and its potential solutions in NB.

**Methods:**

The genetic and clinical data of patients with NB were obtained from the Therapeutically Applicable Research to Generate Effective Treatments dataset. TXNDC17 and BECN1 expressions in NB cells were up‐ and downregulated by transfection with plasmids and shRNA, respectively. Autophagy‐related proteins were detected by western blot. Cell viability was determined using cell proliferation and toxicity experiments. Apoptotic cells were detected using flow cytometry.

**Results:**

Overall, 1076 pediatric and adolescent patients with NB were enrolled in this study. The 10‐year overall survival (OS) rates and event‐free survival (EFS) rates for the patients with a mutation of BECN1 were 37.4 ± 9.1% and 34.5 ± 8.8%, respectively. For patients with a mutation of TXNDC17, the 10‐year OS and EFS were 41.4 ± 5.9% and 24.3 ± 5.1%, respectively, which were significantly lower than those in the unaltered group. The overexpression of BECN1 and TXNDC17 reduced NB sensitivity to cisplatin (DDP), etoposide (VP16), and cyclophosphamide (CTX). Autophagy mediated by BECN1 was regulated by TXNDC17, and this process was involved in the resistance to DDP, VP16, and CTX in NB. Suberoylanilide hydroxamic acid (SAHA) can enhance the sensitivity and apoptosis of NB cells to chemotherapeutics by inhibiting TXNDC17, ultimately decreasing autophagy‐mediated chemoresistance.

**Conclusions:**

Acquired resistance to first‐line chemotherapeutics was associated with autophagy mediated by BECN1 and regulated by TXNDC17, which can be reversed by SAHA.

## 1 Introduction

Neuroblastoma (NB), the most common extracranial solid tumor in children and adolescents, originates from primitive neural crest cells and accounts for approximately 10% of pediatric tumors [1, 2, 3]. By combining various clinical and molecular risk factors, patients with NB can be stratified into low‐, intermediate‐, and high‐risk groups. Unfortunately, even after aggressive multimodal treatment, the 5‐year overall (OS) survival rates for patients with high‐risk NB are still 40%–60% [4, 5, 6].

NB is a chemo‐sensitive tumor; therefore, chemotherapy plays an important role in NB treatment. Induction chemotherapy can effectively reduce tumor burden, create conditions for achieving local control, and guide subsequent treatments [7, 8]. Currently, induction chemotherapy for NB is composed of anthracyclines, platinum, alkylating agents, and topoisomerase II inhibitors [9, 10]. The initial response rate to the induction chemotherapy reaches 80% in different pediatric oncology groups [11, 12]. However, there are still 20% of patients with NB respond poorly to chemotherapy during the treatment due to the acquired drug resistance, and the 5‐year OS rates for these patients are < 20% [8, 13, 14]. Therefore, understanding the mechanism of the acquired chemoresistance of NB may help improve the survival of these patients.

There are many mechanisms resulting in drug resistance in cancer. Autophagy, as a physiological process that occurs in response to metabolic stress, recently is also found to be one of the reasons for chemoresistance [15, 16, 17, 18, 19]. Our previous study confirmed that resistance to cisplatin (DDP), etoposide (VP16), and cyclophosphamide (CTX) in NB cells was promoted by autophagy [20]. Chloroquine (CQ), the most commonly used autophagy inhibitor, can reduce the resistance of tumor cells to chemotherapeutics by blocking the final stage of autophagy [20, 21]. However, CQ did not exert the desired results in clinical trials for cancer treatment [22, 23].

As a major autophagy regulator, BECN1 plays an important role in the formation, elongation, and maturation of the autophagosome [24, 25], and it can be directly regulated by several cellular signals through posttranslational modifications [26, 27]. TXNDC17 is a new member of the thioredoxin family with a molecular mass of 14 kDa and was found to be expressed in multiple types of human tissues [28, 29]. Our previous study showed that BECN1‐mediated autophagy regulated by TXNDC17 is involved in paclitaxel‐induced drug resistance in NB cells [30]. Similar results have been observed in ovarian cancer [31]. However, the roles of TXNDC17 and BECN1 in resistance to first‐line chemotherapeutics in NB are still unclear.

Suberoylanilide hydroxamic acid (SAHA) is a broad‐spectrum hydroxamic histone deacetylase inhibitor (HDACI) that may reduce TXNDC17 expression by upregulating the endogenous suppressor thioredoxin (TRX) [32, 33, 34]. In this study, we explore the role of TXNDC17 in autophagy and its relationship with the resistance to first‐line chemotherapy in NB. We investigate the mechanism by which SAHA regulates autophagy through TXNDC17 and reverses chemoresistance.

## 2 Methods

### 2.1 Patients

The data of patients with NB were selected from the Therapeutically Applicable Research to Generate Effective Treatments (TARGET) dataset using cBioPortal (http://www.cbioportal.org.cn). The selected data included somatic mutations, putative copy number alterations, mRNA expression, and patient clinical data. The *BECN1* and *TXNDC17* mutation rates and types were analyzed using cBioPortal.

### 2.2 Cell Culture

Human NB cell lines SH‐SY5Y (without *MYCN* amplification) and CHP212 (with *MYCN* amplification) were obtained from COBIOER (Nanjing, Jiangsu, China, CBP60743, and CBP60666) and cultured in MEM‐F12 medium supplemented with 10% fetal calf serum (Gibco; Waltham, MA, USA, C11095500BT, C11765500BT, and A3160801). The cells were maintained in a 5% CO_2_ incubator under saturated humidity at 37°C, and cells in the logarithmic growth phase were used for the experiments.

### 2.3 Cell Proliferation and Toxicity Experiment

After digestion and centrifugation, NB cells were seeded in 96‐well plates where each well contained 100 μL culture medium and 1 × 10^4^ cells and each group was repeated in triplicate wells. The plates were incubated overnight to achieve full cell attachment. Subsequently, different doses of the drug were added to wells at the same final medium volume. The Cell Counting Kit‐8 reagent (CCK‐8; APExBIO, Houston, TX, USA, K1018‐100) was diluted into 10% with the MEM medium, and the medium contained drugs was replaced with 100 μL CCK‐8 dilution solution after 24 h of incubation. After incubation for 4 h, the absorbance was measured at 450 nm using a multifunctional microplate detector M200 (Tecan, Zürich, Switzerland), and cell viability was calculated according to the absorbance.

### 2.4 Lentivirus Transfection of Plasmids and shRNA

The TXNDC17 and BECN1 overexpression plasmids, TXNDC17, and BECN1 knockdown shRNAs were purchased from DHbio (Guangzhou, Guangdong, China, EX‐V0925‐Lv105, OENM_003766, SHNM_032731, and SHNM_003766). The pSPAX2 and pMD2.G plasmids were purchased from Youbio (Guangzhou, Guangdong, China, VT1444 and VT1443). The overexpression plasmid and shRNA were mixed with PAX2 and pMD2.G plasmids at a ratio of 10:7.5:3 and transfected into HEK‐293 T cells using Lipofectamine 3000 reagent (Invitrogen, Carlsbad, CA, USA, L3000001). The lentivirus transfection solution was harvested and filtered after 24 and 48 h. NB cells were seeded into 6‐well plates at 60% density in advance, and then 1 mL lentivirus transfection solution, 1 mL culture medium, and an appropriate amount of polybrene were added to each well. After 24 h, the transfection medium was replaced with a fresh medium. The transfected cells were subsequently selected under puromycin with a concentration of 2 μg/mL for 2 weeks.

### 2.5 Flow Cytometry

NB cells were seeded into 6‐well plates at 80% density. After exposure to the drugs for 48 h, the cells were collected and resuspended in the binding buffer to achieve a cell density of 1 × 10^6^ cells/mL. Subsequently, the cells were stained with Annexin V‐FITC/PI reagent (4Abio, Beijing, China, FXP018‐100), and single‐dye tubes were prepared. The samples were analyzed by spectral flow cytometer SP6800 (Sony Biotechnology, San Jose, CA, USA).

### 2.6 Western Blot

Proteins were extracted from tumor cells using RIPA buffer (KeyGEN, Nanjing, Jiangsu, China, KGP2100) and the protein concentration was estimated using the BCA Protein Assay Kit (ThermoFisher, Waltham, MA, USA, 23227). The absorbance was measured at 562 nm using a multifunctional microplate detector M200. Loading buffer was added to the protein at 100°C for 10 min to denature the protein, 20 μg denatured protein from each group was separated by SDS‐PAGE. The separated proteins were transferred onto PVDF membranes and blocked with skim milk powder for 2 h. The primary antibody was added at an appropriate dilution and the blots were incubated overnight. Subsequently, the blots were rinsed with TBS‐T three times for 15 min each and incubated with the secondary antibody for 1 h. After rinsing with TBS‐T, the blots were stained with enhanced chemiluminescence and visualized using a chemiluminescence imaging system (Bio‐Rad Laboratories, Hercules, CA, USA). Primary antibodies to BECN1, LC3B, and P62 were purchased from Abclonal (Wuhan, Hubei, China, A10101, A19665, and A19700), and the antibody to TXNDC17 was purchased from Bioworld (Shanghai, China, BS62549‐2). HRP‐labeled secondary anti‐mouse and anti‐rabbit antibodies were purchased from FDbio (Hangzhou, Zhejiang, China, FDM007, and FDR007). Glyceraldehyde 3‐phosphate dehydrogenase (GAPDH) was used as a reference in this study.

### 2.7 Statistical Analysis

Event‐free survival (EFS) was defined as the time between diagnosis and progression, relapse, second malignancy, death from any cause, or last follow‐up contact for patients who did not experience any event. Overall survival (OS) was defined as the time between diagnosis and death from any cause or the last follow‐up visit for live patients. OS and EFS were estimated using the Kaplan–Meier method, and the log‐rank test was used to compare the risk of adverse events between the groups. Correlations between factors were analyzed by Spearman correlation analysis, and cell viability and apoptosis rates between different groups were assessed by Student's *t*‐test. P values from 0.01 to 0.05, from 0.001 to 0.01, or < 0.001 were considered significant (*), very significant (**), or highly significant (***), respectively. Statistical analysis was carried out using SPSS Statistics 25 (IBM, Armonk, NY, USA) and GraphPad Prism (version 10.0; GraphPad Software, San Diego, CA, USA).

## 3 Results

### 3.1 The Mutation of *BECN1* and *TXNDC17* in Patients With NB Was Associated With Poor Prognosis

**FIGURE 1:**
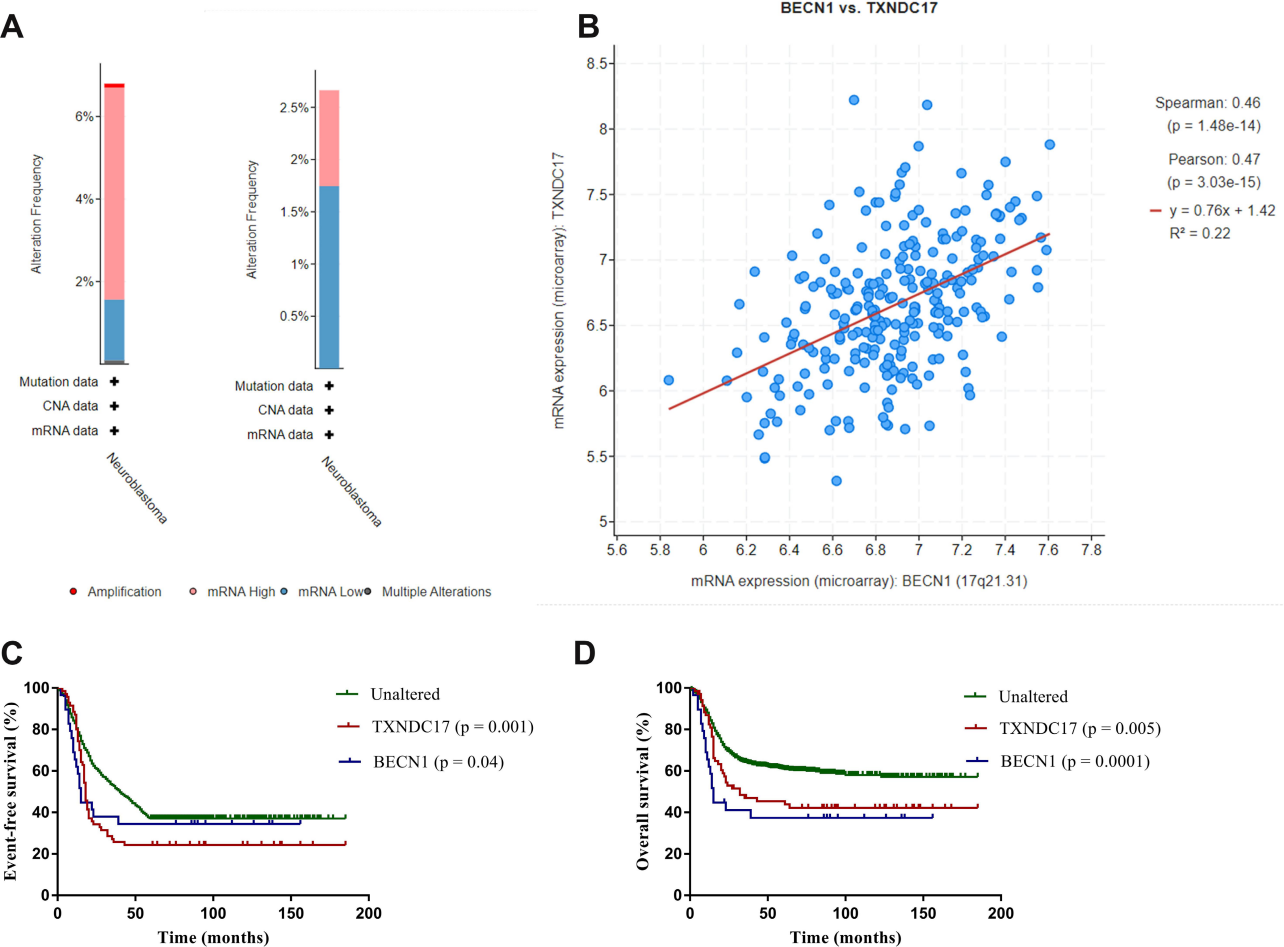
Prognosis for patients with a mutation of BECN1 or TXNDC17. (A) Mutation frequency and specific mutation types of *BECN1* and *TXNDC17* in the sample data. (B) Correlation analysis between mRNA expression of *BECN1* and *TXNDC17*. (C) Patients with mutation of *BECN1* (*p* = 0.001) or *TXNDC17* (*p* = 0.04) have lower 10‐year EFS rates compared to the unaltered group. (D) Patients with mutation of *BECN1* (*p* = 0.005) or *TXNDC17* (*p* = 0.0001) have lower 10‐year OS rates compared to the unaltered group.

In total, 1076 children and adolescents with NB selected from the TARGET dataset using cBioPortal were enrolled in this study. The inclusion criteria were patients with a diagnosis of NB between 2003 and 2018, age < 18 years, and clinical data uploaded to the TARGET dataset. The median age of the patients was 8.6 years, and the male‐to‐female ratio was 1.3:1. The detailed clinical features are displayed in Table 1. Patients were enrolled in clinical trials, including ANBL00B1, ANBL0532, ANBL0032, and AEPI07N1. The commonly used induction chemotherapy regimens included CTX, doxorubicin, and vincristine, alternated with DDP and VP16. The 10‐year EFS rate of the whole group was 36.9% ± 1.5%, and the 10‐year OS rate was 57.5% ± 1.8%. The mutation frequencies of *BECN1* and *TXNDC17* were 6.80% and 2.66%, respectively (Figure 1A). The mRNA expression of *BECN1* was associated with *TXNDC17* (*p* = 1.48 × 10^−14^), and the Spearman correlation coefficient r was 0.46 (Figure 1B). The 10‐year EFS rates for the patients with no mutations of *BECN1* and *TXNDC17* (*n* = 984), mutation of *BECN1* (*n* = 70), and mutation of *TXNDC17* (*n* = 29) were 37.1% ± 1.5%, 34.5% ± 8.8%, and 24.3% ± 5.1%, respectively (*p* < 0.05, Figure 1C); the 10‐year OS rates were 58.0% ± 1.8%, 37.4% ± 9.1%, and 41.4% ± 5.9%, respectively (*p* < 0.05, Figure 1D).

**TABLE 1:** Characteristics of the patients with neuroblastoma.

Characteristics	Cases (*n*)	Percent (%)
Sex		
Male	613	57
Female	463	43
Race		
White	792	74
Others	284	26
Ethnicity		
Not Hispanic or Latino	870	81
Hispanic or Latino	206	19
INSS stage		
Stage I	89	8
Stage II	61	6
Stage III	94	9
Stage IV	777	72
Stage IVS	55	5
MYCN status		
Amplified	300	28
Not amplified	760	71
Unknown	16	1
Primary site		
Abdomen	827	77
Pelvic	15	1
Chest	106	10
Head and neck	21	2
Others	107	10

Abbreviation: INSS, International Neuroblastoma Staging System.

### 3.2 Upregulation of BECN1 or TXNDC17 Resulted in Decreased Sensitivity to DDP, VP16, and CTX in NB

**FIGURE 2:**
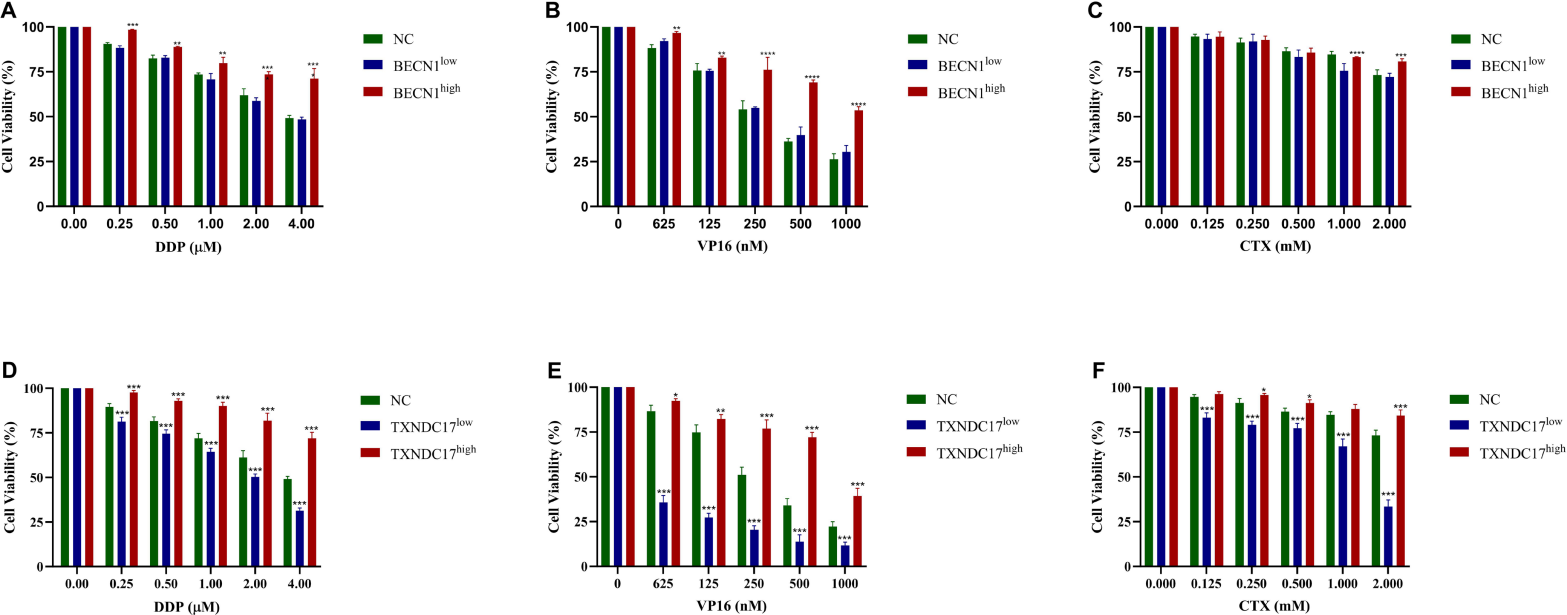
Upregulation of BECN1 or TXNDC17 results in decreased chemosensitivity in NB cells. Upregulation of BECN1 results in decreased chemosensitivity and downregulation of BECN1 led to increased chemosensitivity in NB cells exposed to DDP (A), VP16 (B), and CTX (C). Upregulation of TXNDC17 results in decreased chemosensitivity and downregulation of TXNDC17 leads to increased sensitivity in NB cells exposed to DDP (D), VP16 (E), and CTX (F). *p < 0.05, **p < 0.01, ***p < 0.001, two‐sided *t*‐test.

In our previous study, we demonstrated that resistance to DDP, VP16, and CTX in NB was induced by autophagy, and this process was associated with BECN1 [20]. BECN1^high^, BECN1^low^, TXNDC17^high^, and TXNDC17^low^ SH‐SY5Y cells were obtained by up‐ and downregulating BECN1 and TXNDC17. Negative control (NC), BECN1^high^, BECN1^low^, TXNDC17^high^, and TXNDC17^low^ cells were exposed to DDP, VP16, and CTX at the same concentration for the same time. The respective cell proliferation rates of BECN1^high^, NC, and BECN1^low^ were 74% versus 62% versus 59% after exposure to 2 μM DDP, 76% versus 54% versus 55% with 250 nM VP16, and 81% versus 73% versus 72% with 2 mM CTX (Figure 2A–C). These results show that BECN1 overexpression results in lower sensitivity to DDP, VP16, and CTX in NB cells. While the respective cell proliferation rates of TXNDC17^high^, NC, and TXNDC17^low^ were 82% versus 61% versus 50% with 2 μM DDP, 77% versus 51% versus 20% with 250 nM VP16, and 84% versus 73% versus 33% with 2 mM CTX (Figure 2D–F). These results indicated that the upregulation of TXNDC17 also reduced the sensitivity of NB cells to DDP, VP16, and CTX.

### 3.3 BECN1‐Mediated Autophagy Was Regulated by TXNDC17 in NB

**FIGURE 3:**
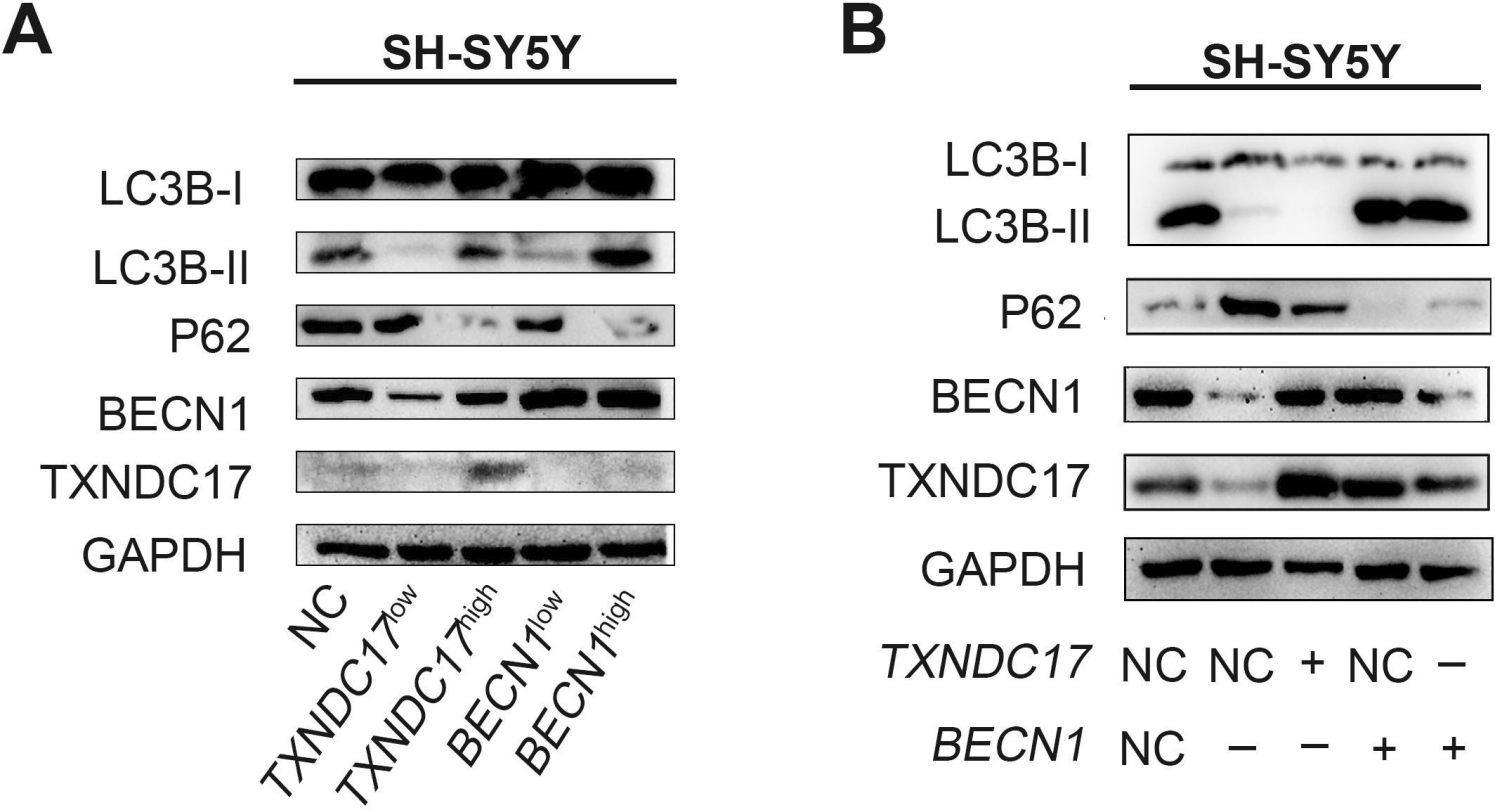
BECN1‐mediated autophagy is regulated by TXNDC17 in NB. (A) Level of LC3B‐II decreases, and that of P62 increases after the downregulation of BECN1. The level of LC3B‐II increases and that of P62 decreases after the upregulation of BECN1. The level of LC3B‐II decreases, and that of P62 increases after the downregulation of TXNDC17. The level of LC3B‐II increases, and P62 decreases after the upregulation of TXNDC17. (B) Compared with BECN1^high^ cells, the levels of BECN1, LC3B‐II, and TXNDC17 decrease, and that of P62 increases in BECN1^high^TXNDC17^low^ cells. Compared with BECN1^low^ cells, the levels of BECN1, LC3B‐II, and TXNDC17 increase, and that of P62 decreases in BECN1^low^TXNDC17^high^ cells.

The levels of autophagy‐related proteins in BECN1^high^ and BECN1^low^ cells were detected by western blot, and the results showed that the levels of LC3B‐II increased and those of P62 decreased, indicating that autophagy was enhanced in BECN1^high^ cells. However, the levels of LC3B‐II decreased, and P62 increased, indicating that autophagy was decreased in BECN1^low^ cells (Figure 3A). These results suggested that autophagy in NB was mediated by BECN1. Western blot was performed to detect autophagy‐related proteins in the TXNDC17^high^ and TXNDC17^low^ cells. The results showed high levels of LC3B‐II and low levels of P62 in the TXNDC17^high^ cells, indicating enhanced autophagy. The levels of LC3B‐II decreased, and P62 increased in TXNDC17^low^ cells, indicating that autophagy was inhibited (Figure 3A). These results show that TXNDC17 is involved in the regulation of autophagy in NB cells and interacts with BECN1.

To further clarify the relationship between TXNDC17 and BECN1, BECN1^high^TXNDC17^low^, and BECN1^low^TXNDC17^high^ cells were obtained by downregulating TXNDC17 in BECN1^high^ cells and upregulating TXNDC17 in BECN1^low^ cells. Compared to BECN1^high^ cells, the levels of BECN1, LC3B‐II, and TXNDC17 were lower, and P62 was higher in BECN1^high^TXNDC17^low^ cells. Compared to BECN1^low^ cells, the levels of BECN1, LC3B‐II, and TXNDC17 were higher, and P62 was lower in BECN1^low^TXNDC17^high^ cells (Figure 3B). These results indicate that BECN1‐mediated autophagy is regulated by TXNDC17 in NB.

### 3.4 SAHA Inhibited Autophagy by Decreasing the Expression of TXNDC17

**FIGURE 4:**
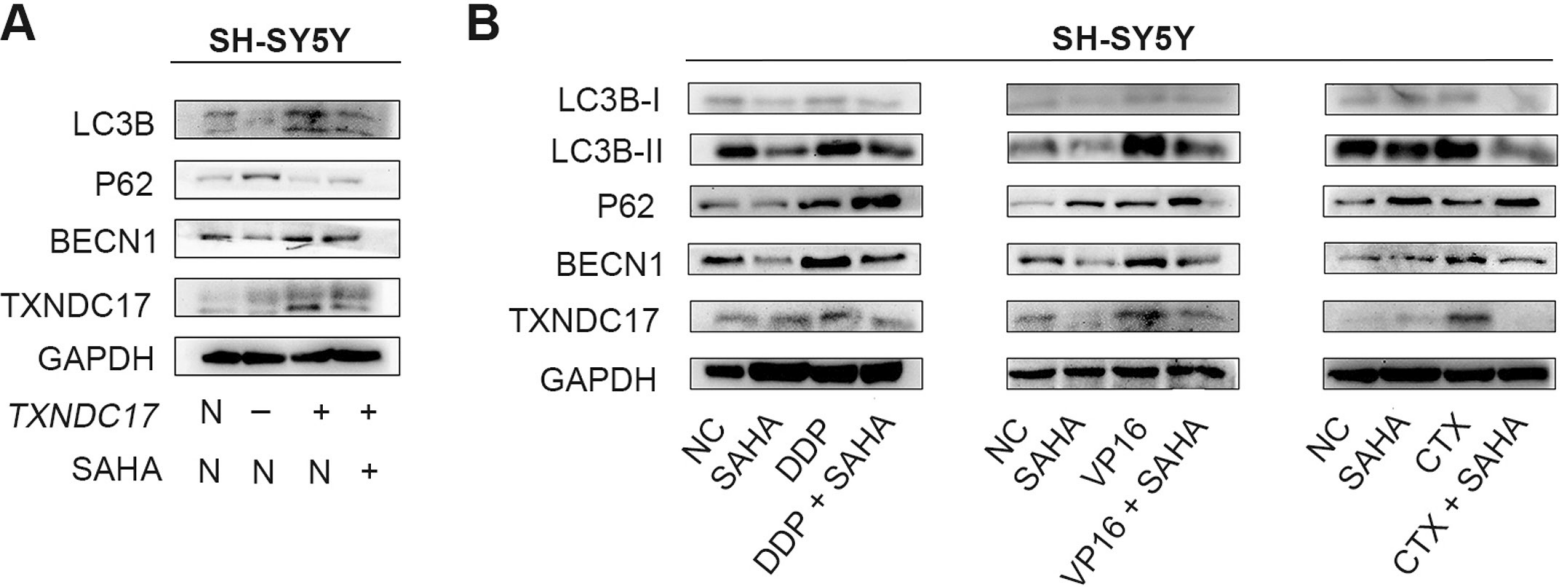
SAHA inhibits autophagy by decreasing the expression of TXNDC17. (A) SH‐SY5Y cells with upregulated TXNDC17 show decreased LC3B‐II, TXNDC17, and BECN1 levels and increased P62 after exposure to SAHA. (B) Compared with using DDP, VP16, and CTX alone, the levels of LC3B‐II, TXNDC17, and BECN1 decrease, and that of P62 increases after combining SAHA.

The IC50 of SH‐SY5Y cells to SAHA was 2 μM, which was detected by the Cell Counting Kit‐8 method. TXNDC17^high^ and NC cells were exposed to 1 μM SAHA for 48 h, and a western blot was used to detect autophagy‐related proteins. Compared to the NC group, the levels of LC3B‐II, TXNDC17, and BECN1 decreased, and P62 increased in TXNDC17^high^ cells after exposure to SAHA (Figure 4A). SAHA effectively inhibited the expression of TXNDC17 and then inhibited autophagy. SH‐SY5Y cells were exposed to DDP, VP16, or CTX, with or without SAHA, for 48 h. The levels of LC3B‐II, TXNDC17, and BECN1 decreased in the group treated with SAHA (Figure 4B). This suggests that DDP, VP16, and CTX combined with SAHA can effectively inhibit autophagy‐induced chemoresistance.

### 3.5 SAHA Enhanced the Sensitivity of NB to DDP, VP16, and CTX

**FIGURE 5:**
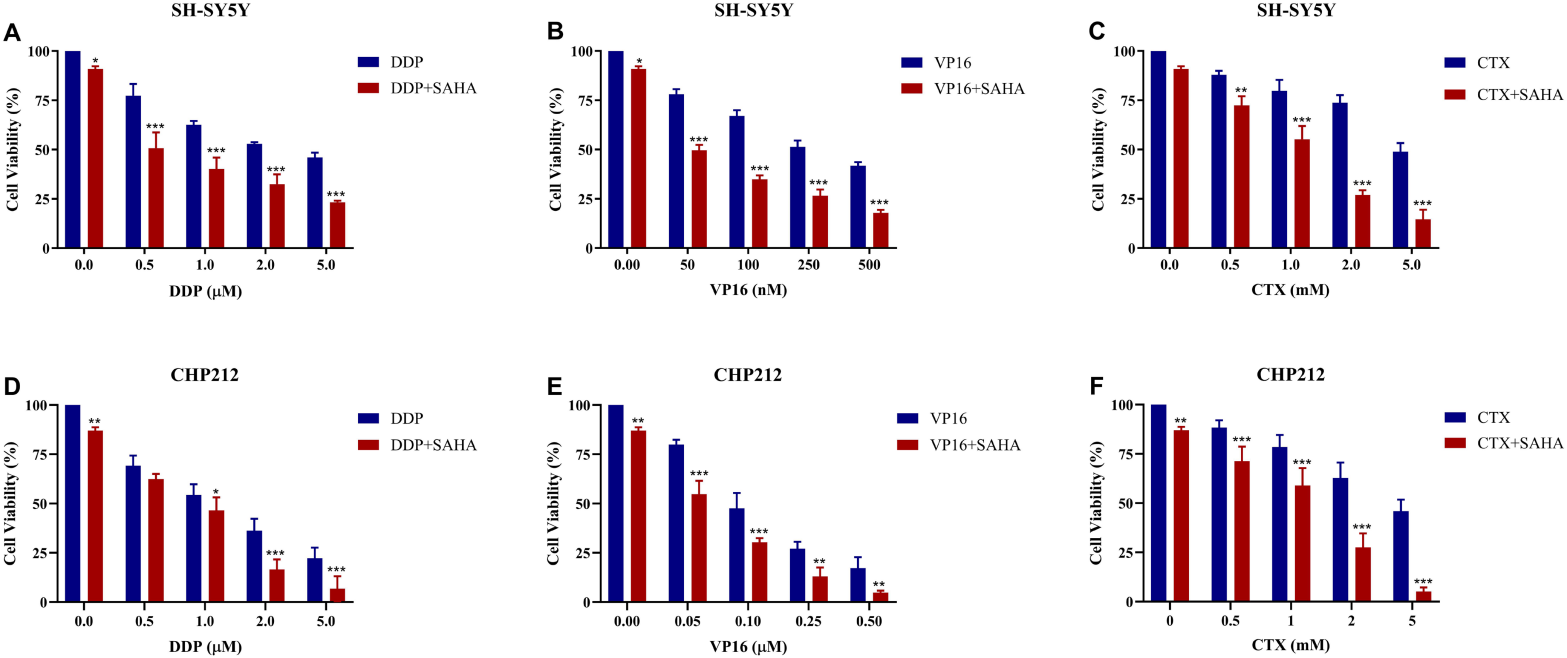
SAHA can enhance the sensitivity of NB to chemotherapy. (A) Compared with using DDP alone, the cell proliferation rate of SH‐SY5Y is significantly reduced after combining SAHA. (B) Compared with using VP16 alone, the cell proliferation rate of SH‐SY5Y is significantly reduced after combining SAHA. (C) Compared with using CTX alone, the cell proliferation rate of SH‐SY5Y is significantly reduced after combining SAHA. (D) Compared with using DDP alone, the cell proliferation rate of CHP212 is significantly reduced after combining SAHA. (E) Compared with using VP16 alone, the cell proliferation rate of CHP212 is significantly reduced after combining SAHA. (F) Compared with using CTX alone, the cell proliferation rate of CHP212 is significantly reduced after combining SAHA. *p < 0.05, **p < 0.01, ***p < 0.001, two‐sided *t*‐test.

To determine the effects of SAHA combined with chemotherapeutics, SH‐SY5Y and CHP212 cells were exposed to different concentrations of DDP, VP16, and CTX with or without the addition of 0.5 μM SAHA. The proliferation rate of SH‐SY5Y and CHP212 cells exposed to SAHA alone was 90% and 87%, while SH‐SY5Y cells treated with DDP, VP16, or CTX alone showed proliferation rates of 53%, 51%, and 74%, respectively. The cell proliferation rates after combination treatment with SAHA were 32%, 27%, and 27%, respectively. Similar results were also observed in CHP212. This indicates that SAHA could only slightly inhibit the growth of tumor cells when used alone, and the inhibitory effect of chemotherapeutics was significantly enhanced when combined with SAHA (Figure 5A–F). SAHA enhanced the sensitivity of NB cells to DDP, VP16, and CTX.

### 3.6 SAHA Enhanced Chemotherapy‐Induced Apoptosis in NB

**FIGURE 6:**
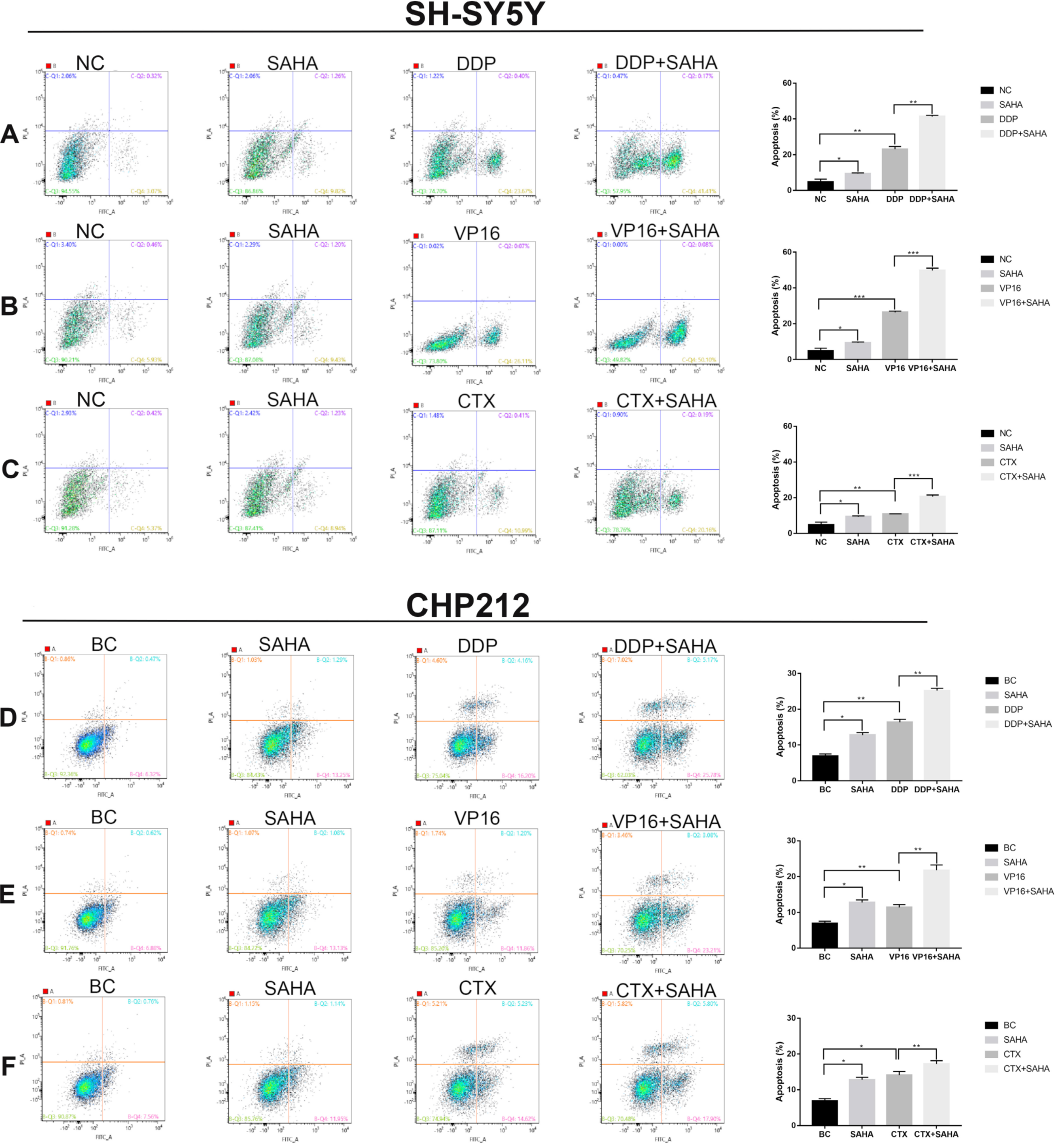
SAHA can enhance chemotherapy‐induced apoptosis in NB. (A) Compared with exposure to DDP alone, the apoptosis rate of SH‐SY5Y is enhanced in the DDP + SAHA group. (B) Compared with exposure to VP16 alone, the apoptosis rate of SH‐SY5Y is enhanced in the VP16 + SAHA group. (C) Compared with exposure to CTX alone, the apoptosis rate of SH‐SY5Y is enhanced in the CTX + SAHA group. (D) Compared with exposure to DDP alone, the apoptosis rate of CHP212 is enhanced in the DDP + SAHA group. (E) Compared with exposure to VP16 alone, the apoptosis rate of CHP212 is enhanced in the VP16 + SAHA group. (F) Compared with exposure to CTX alone, the apoptosis rate of CHP212 is enhanced in the CTX + SAHA group. *p < 0.05, **p < 0.01, ***p < 0.001, two‐sided *t*‐test.

To further explore the additional mechanisms of SAHA combined with chemotherapeutics in killing NB cells, SH‐SY5Y and CHP212 cells were exposed to 5 μM DDP, 500 nM VP16, and 5 mM CTX with or without the addition of 0.5 μM SAHA for 48 h. Apoptosis was analyzed using flow cytometry. The apoptotic rates of SH‐SY5Y cells exposed to DDP, VP16, or CTX alone were 23.1%, 26.4%, and 11.0%, respectively. The apoptosis rates increased to 41.4%, 50.0%, and 20.6%, respectively, after combination treatment with SAHA. Similar results were also observed in CHP212. Compared to treatment with chemotherapeutics or SAHA alone, cell apoptosis was significantly enhanced after combination treatment with SAHA (Figure 6A–F). These results indicate that SAHA also enhances the apoptosis of NB, in addition to improving the sensitivity to chemotherapeutics.

## 4 Discussion

This study investigated the mechanism of drug resistance to first‐line chemotherapeutics in NB through bioinformatics and basic experiments and found that acquired resistance to first‐line chemotherapeutics was associated with autophagy mediated by BECN1 and regulated by TXNDC17, which can be reversed by SAHA.

Autophagy, as a pathway of cellular tolerance to stress that is associated with several physiological and pathological states, such as intracellular circulation, and nutrient starvation, was found to play an important role in drug resistance in cancer [35, 36, 37, 38, 39, 40]. In several tumor types, autophagy protects tumor cells against stress, leading to chemoresistance [41, 42, 43]. Our previous studies also found that autophagy induces resistance to paclitaxel, DDP, VP16, and CTX in NB [20, 30]. However, the underlying mechanism is still unclear.

To further clarify the relationship between autophagy and chemoresistance in NB, the autophagy main regulator BECN1 was down‐ and upregulated in this research. The results showed that the overexpression of BECN1 enhanced autophagy and significantly reduced the sensitivity of NB cells to DDP, VP16, and CTX. Enhanced BECN1‐mediated autophagy is one of the reasons for the resistance to DDP, VP16, and CTX. Previous studies have shown that BECN1‐mediated autophagy is associated with leukemia resistance to CTX [44]; lung cancer, embryonal carcinoma, cervical carcinoma resistance to DDP [45, 46, 47]; and cervical cancer resistance to VP16 [48]. These findings suggest that BECN1‐mediated autophagy is a common factor in chemotherapy resistance, thus adversely affecting the prognosis of multiple tumor types, including NB.

To confirm the effect of BECN1 in patients with NB, we performed a survival analysis of 1076 patients with NB enrolled in the TARGET public database and found that the 10‐year EFS and OS rates of patients with *BECN1* mutations were poorer than those of patients without *BECN1* mutations. BECN1 was involved in the regulation of the occurrence and development of NB and had a similar effect in protecting and promoting the growth of tumor cells.

However, BECN1 is regulated by several cellular signals that ultimately regulate autophagy [27, 49, 50]. In the survival analysis of the enrolled patients, the 10‐year EFS and OS rates were significantly lower in the *TXNDC17* mutation group. Both TXNDC17 and BECN1 are significantly involved in the chemoresistance of NB. A Spearman correlation analysis revealed that the mRNA expression levels of *TXNDC17* and *BECN1* were positively correlated. Our previous study showed that TXNDC17 is associated with primary resistance to paclitaxel in NB [30]. Moreover, TXNDC17 promotes tumor resistance to paclitaxel in ovarian cancer by regulating BECN1‐mediated autophagy, and high TXNDC17 expression has been confirmed to be associated with poor prognosis in patients with ovarian cancer [31].

To further explore the relationship between TXNDC17 and BECN1 in autophagy‐related chemoresistance, TXNDC17 was down‐ and upregulated using genetic engineering. BECN1 levels decreased and autophagy was inhibited after TXNDC17 downregulation. In validation experiments, autophagy in downregulated BECN1 NB cells was enhanced by upregulating TXNDC17. Thus, TXNDC17 regulates autophagy by regulating BECN1 expression in NB.

Previous studies have shown that the transcription factor NF‐κB drives the expression of BECN1 [51], while TXNDC17 inhibits tumor necrosis factor‐induced NF‐κB activation. DYNLL1, which was the only identified potential substrate of TXNDC17, could interact with the inhibitors of NF‐κB [52]. In addition, the NF‐κB1 family member RELA upregulated the mRNA and protein levels of BECN1 in different cellular systems through the activation of a conserved NF‐κB1 binding site in the promoter of the *BECN1* gene [53]. Therefore, we hypothesized that TXNDC17 regulates BECN1‐mediated autophagy through the NF‐κB pathway. This study also found that the overexpression of TXNDC17 can reduce the sensitivity of NB cells to DDP, VP16, and CTX, leading to drug resistance, which has an effect similar to that of BECN1.

As a broad‐spectrum HDACI, SAHA upregulates the expression of TRX inhibitors in various tumor cells, thereby inhibiting TRX activity [34, 54, 55]. TXNDC17 is a member of the TRX family. This study showed that TXNDC17, along with its related autophagy, was specifically inhibited by SAHA in NB. After the combination of chemotherapeutics and SAHA, autophagy decreased, and NB was significantly more sensitive to DDP, VP16, and CTX. These results suggest that autophagy‐induced chemoresistance can be reversed by combining SAHA with chemotherapy.

Moreover, this study found that SAHA can promote the apoptosis of NB cells, which again proves that SAHA has multiple antitumor effects on NB. However, the cell inhibition rate was much greater than the apoptosis rate in NB cells exposed to the same concentration and treatment time as the chemotherapeutics, indicating that apoptosis accounted for only a small proportion of the killing activity of SAHA. Therefore, we considered this to have little impact on the conclusions drawn from this study. Whether there is a synergy between the molecular mechanisms of autophagy and apoptosis is worth further investigation.

In conclusion, this study confirmed that enhanced BECN1‐mediated autophagy was related to the resistance of NB cells to DDP, VP16, and CTX in a first‐line induction chemotherapy regimen. SAHA reduces BECN1 expression by inhibiting TXNDC17, thereby reversing chemotherapy resistance and improving the sensitivity of NB cells to chemotherapy. These findings should be investigated further to improve the efficacy of NB treatment.

## Author Contributions

**Chenggong Zeng:** writing – original draft, methodology, writing – review and editing, software, formal analysis, data curation. **Zhuoran Li:** validation, methodology, visualization, software, formal analysis, data curation, resources. **Zhiqing Wei:** methodology, validation, visualization, software, formal analysis, data curation, resources. **Tingting Chen:** methodology, validation, visualization, software, formal analysis, supervision. **Juan Wang:** supervision, investigation. **Junting Huang:** investigation, supervision. **Feifei Sun:** supervision, investigation. **Jia Zhu:** investigation, supervision. **Suying Lu:** investigation, supervision. **Zijun Zhen:** conceptualization, investigation, funding acquisition, validation, project administration, supervision.

## Ethics Statement

This study has no ethics approval to declare.

## Conflicts of Interest

The authors declare no conflicts of interest.

## Data Availability

The data that support the findings of this study are available from the corresponding author upon reasonable request.
